# Systematic Review of Management Strategies for Alport Syndrome: Implications for Male Patients

**DOI:** 10.1002/hsr2.70595

**Published:** 2025-03-30

**Authors:** Zouina Sarfraz, Ayesha Khan, Maryyam Liaqat, Aden Khan, Faheem Javad, Meher Saleem, Azza Sarfraz, Musfira Khalid, Muzna Sarfraz, Manish Kc, Omar Irfan

**Affiliations:** ^1^ Fatima Jinnah Medical University Lahore Pakistan; ^2^ Dow International Medical College Karachi Pakistan; ^3^ King Edward Medical University Lahore Pakistan; ^4^ Al‐Nafees Medical College Islamabad Pakistan; ^5^ The Aga Khan University Karachi Pakistan; ^6^ KIST Medical College Lalitpur Nepal

**Keywords:** Alport syndrome, chronic disease management, genetic disorders, medical innovation, patient outcomes, renal health, systemic manifestations, therapeutic strategies

## Abstract

**Background and Aims:**

Alport Syndrome (AS) is a rare genetic disorder characterized by progressive kidney disease, hearing loss, and ocular abnormalities, with an incidence of approximately 1 in 50,000 newborns. Due to the severity of the disease, particularly in males with X‐linked inheritance, this systematic review consolidates current management strategies, highlighting advancements and existing gaps in treatment options.

**Methods:**

This systematic review followed a protocol registered on the OSF platform (osf. io/k86ms). A comprehensive search of PubMed, Web of Science, Scopus, Cochrane Library, Embase, ClinicalTrials.gov, and the WHO ICTRP was completed by December 24, 2023. Studies eligible for inclusion were clinical trials or observational studies evaluating AS management. Four clinical trials from six publications and two observational studies met the inclusion criteria. The risk of bias was assessed using the Cochrane ROB 2 tool for clinical trials and the Newcastle–Ottawa Scale (NOS) for observational studies. Key interventions examined included bardoxolone methyl, ramipril, and losartan.

**Results:**

Bardoxolone methyl, ramipril, and losartan demonstrated potential benefits in slowing renal disease progression in AS. Observational studies indicated that early intervention might delay the need for dialysis and improve life expectancy. However, significant heterogeneity among studies precluded quantitative synthesis. Ongoing studies on AS management encompass 25 trials involving 52,135 participants, reflecting an active area of research.

**Conclusion:**

Bardoxolone methyl, ramipril, and losartan show promise in delaying renal failure in AS. Nonetheless, the findings highlight the critical need for larger, more diverse trials to validate these therapies and explore additional treatment strategies. Future research must aim to address these evidence gaps, improving treatment efficacy and patient quality of life, particularly for males disproportionately affected by the disease.

****Protocol** Registration:**

The protocol for this systematic review is registered in the Open Science Framework (OSF): osf. io/k86ms.

## 1 Introduction

Alport syndrome (AS) is a rare genetic disorder characterized by progressive kidney disease, sensorineural hearing loss, and ocular abnormalities [1]. It is caused by mutations in the genes encoding Type IV collagen, which is essential for the proper functioning of the glomerular basement membrane in kidneys, cochlea, and eye structures [2]. AS affects approximately 1 in 50,000 live births and is a significant cause of end‐stage renal disease (ESRD) in young adults [1]. The progressive nature of the disease and its impact on multiple organ systems severely affect patients' quality of life (QoL), necessitating comprehensive care [3]. Males with X‐linked AS typically have more severe manifestations compared to females. Understanding available and upcoming healthcare strategies is crucial to developing effective treatment plans, improving patient outcomes, and offering a more comprehensive approach to managing the disease.

X‐linked AS is the most common form, affecting 80% of AS patients, caused by mutations in the COL4A5 gene 2. Males with X‐linked AS generally have more severe symptoms and earlier disease onset than females. Autosomal recessive AS impacts 15% of patients, resulting from mutations in the COL4A3 or COL4A4 genes [4]. Both males and females are affected equally, with disease severity similar to X‐linked AS. The rarest form, autosomal dominant AS, accounts for 5% of cases, caused by mutations in COL4A3 or COL4A4 genes. Both genders are affected equally, but the disease is generally milder than the X‐linked and autosomal recessive forms [5]. Mutations in the COL4A3, COL4A4, and COL4A5 genes disrupt the formation of the collagen IV network, leading to the clinical manifestations of AS [5]. Identification of these mutations allows for a more accurate diagnosis and tailored management strategies.

Clinical manifestations include progressive renal disease, characterized by hematuria, proteinuria, and eventual development of ESRD [6, 7]. Males with AS often have a faster progression to renal failure compared to females [8, 9]. Auditory indicators include sensorineural hearing loss, typically affecting high‐frequency sounds and becoming apparent in late childhood or adolescence. This complication significantly impacts the QoL and daily functioning of affected individuals. Ocular presentations comprise anterior lenticonus, dot‐and‐fleck retinopathy, and corneal opacities, which can lead to visual impairments and necessitate regular ophthalmologic evaluations. Extrarenal manifestations include leiomyomatosis and, rarely, cardiovascular abnormalities. These manifestations may require additional management and monitoring.

In addition to these well‐characterized symptoms, recent findings suggest that AS can also present with rare oral manifestations, specifically mucous or bullous pemphigoid. These conditions are chronic immunologically mediated bullous disorders that affect the mucosal surfaces, including the oral cavity, and are characterized by subepithelial blister formation due to autoantibodies binding to antigens in the basement membrane zone. Notably, an interesting case was reported where a patient with AS exhibited mucous membrane pemphigoid after end‐stage kidney disease and kidney transplantation [10]. Furthermore, bullous pemphigoid has been identified in a Japanese patient undergoing hemodialysis due to X‐linked AS, underscoring the potential for these atypical manifestations in patient groups [11].

Diffuse leiomyomatosis affecting the tracheobronchial tree and esophagus has been documented in certain familial cases of Alport syndrome [12]. Typically manifesting in later childhood, the symptoms of this condition can include difficulties swallowing (dysphagia), vomiting after meals, repeated episodes of bronchitis, pain located beneath the sternum or in the upper stomach area, breathing difficulties, noisy breathing (stridor), and coughing. This manifestation adds another layer to the diverse clinical spectrum associated with AS, highlighting its potential to affect multiple organ systems beyond the kidneys [1, 13].

Diagnostic approaches include genetic testing such as the identification of pathogenic mutations in the COL4A3, COL4A4, or COL4A5 genes, allowing for definitive diagnosis and guiding therapeutic decisions [13]. Renal biopsy evaluates renal tissue for characteristic AS findings, such as irregular thickening and thinning of the glomerular basement membrane. This invasive procedure is particularly useful when genetic testing is inconclusive. Detection of ocular and auditory manifestations enables early intervention and management of complications [14].

Treatment strategies include renin‐angiotensin‐aldosterone system (RAAS) blockade with angiotensin‐converting enzyme (ACE) inhibitors (i.e., ramipril and enalapril to delay the progression of renal disease by reducing proteinuria and maintaining kidney function) and angiotensin receptor blockers (ARBs) (i.e., losartan to slow down renal disease progression, with better tolerance in differential patient groups) [15]. Supportive care such as dietary modifications with low‐salt and low‐protein diets, management of hypertension, anemia, bone disease, and fluid overload through targeted therapies and lifestyle modifications improve the overall QoL. Potential future treatment using gene editing or gene replacement strategies to correct the underlying genetic defect, providing a more targeted and potentially curative approach [16]. Research is ongoing to investigate the use of stem cells to regenerate damaged kidney tissue and restore function, offering a promising alternative to dialysis or transplantation for AS patients.

Timely identification and initiation of appropriate treatments are crucial to slow disease progression and minimize complications. Personalized medicine may also bolster the understanding of the genetic and molecular underpinnings of AS through targeted and effective therapies tailored to individual patients. Comprehensive care that addresses not only the renal manifestations of AS but also the auditory, ocular, and extrarenal complications is essential for improving the overall well‐being of affected individuals.

The aim of this study is to systematically review clinical trials and observational cohorts, along with records of all registered trials of AS, to assess the clinical efficacy, effectiveness, safety and QoL of different management approaches, with implications for males.

## 2 Methods

This study is reported per the PRISMA Statement 2020 Guidelines [17]. This study analyzes existing literature, does not involve primary data collection/interaction with human participants, and therefore, is exempt from obtaining ethical approval. The systematic review poses no potential risks or harm to any subjects, adhering to Helsinki guidelines. The protocol for this systematic review is registered in the Open Science Framework (OSF) platform (osf. io/k86ms).

### 2.1 Search Strategy

A comprehensive search strategy was executed covering a timeline of inception until December 24, 2023, using the following databases: PubMed, Web of Science, Scopus, Cochrane Library, and Embase. Additionally, ClinicalTrials.gov and the WHO International Clinical Trials Registry Platform (ICTRP) were searched for registered trials of AS. A combination of keywords was used to search these databases and trial websites, as presented in Table 1.

**Table 1:** Keyword combinations for the searched databases/trial registries.

Database	Keywords
PubMed	**Alport Syndrome:** “nephritis, hereditary”[MeSH Terms] OR (“nephritis”[All Fields] AND “hereditary”[All Fields]) OR “hereditary nephritis”[All Fields] OR (“alport”[All Fields] AND “syndrome”[All Fields]) OR “alport syndrome”[All Fields] **Clinical Trial:** “clinical trial”[Publication Type] OR “clinical trials as topic”[MeSH Terms] OR “clinical trial”[All Fields] **Healthcare:** “delivery of health care”[MeSH Terms] OR (“delivery”[All Fields] AND “health”[All Fields] AND “care”[All Fields]) OR “delivery of health care”[All Fields] OR “healthcare”[All Fields] OR “healthcare's”[All Fields] OR “healthcares”[All Fields] **Pharmacological Treatments:** “drug therapy”[MeSH Terms] OR (“drug”[All Fields] AND “therapy”[All Fields]) OR “drug therapy”[All Fields] OR (“pharmacological”[All Fields] AND “treatments”[All Fields]) OR “pharmacological treatments”[All Fields] **Lifestyle:** “life style”[MeSH Terms] OR (“life”[All Fields] AND “style”[All Fields]) OR “life style”[All Fields] OR “lifestyle”[All Fields] OR “lifestyles”[All Fields] **Supportive:** “support”[All Fields] OR “support's”[All Fields] OR “supported”[All Fields] OR “supporter”[All Fields] OR “supporter's”[All Fields] OR “supporters”[All Fields] OR “supporting”[All Fields] OR “supportive”[All Fields] OR “supportiveness”[All Fields] OR “supports”[All Fields]
Web of Science	(“Alport Syndrome” AND (“clinical trial” OR “healthcare approaches” OR “pharmacological treatments” OR “lifestyle modifications” OR “supportive care” OR “males” OR “intervention” OR “therapy” OR “management” OR “outcomes” OR “quality of life” OR “survival rates”))
Scopus	(“Alport Syndrome” AND (“treatment” OR “clinical trial” OR “drug therapy” OR “nonpharmacological intervention” OR “patient care” OR “prognosis” OR “renal disease”))
Cochrane Library	(“Alport Syndrome” AND (“randomized controlled trial” OR “systematic review” OR “meta‐analysis” OR “observational cohort” OR “patient outcomes” OR “symptom management”))
Embase	(“Alport Syndrome” AND (“clinical study” OR “therapeutics” OR “renal management” OR “kidney function” OR “disease progression” OR “renal outcomes”))
ClinicalTrials.gov and WHO ICTRP	(“Alport Syndrome” OR “Alport's Syndrome” OR “Hereditary Nephritis”) AND (clinical trial OR cohort OR observational OR interventional) AND (male)

### 2.2 Eligibility Criteria for Inclusion of Completed Studies

- P (Population): Studies focusing on individuals of all age groups with a confirmed diagnosis of any type of AS were included. Studies that did not specify the gender of participants or included both male and female patients were also considered if they provided disaggregated data for male patients.
- I (Intervention): Studies evaluating various management approaches, including pharmacological treatments (e.g., ACE inhibitors, angiotensin receptor blockers, etc.), lifestyle modifications (e.g., dietary changes, exercise interventions), and supportive care (e.g., dialysis, renal transplantation) were eligible for inclusion.
- C (Comparison): Eligible studies were required to compare outcomes between different healthcare approaches and their effects on AS.
- O (Outcomes): Studies reporting on any relevant clinical outcomes including efficacy, effectiveness, safety and QoL were considered for inclusion.
- S (Study Design): Clinical trials and observational cohort studies, along with records of all registered trials of AS, were considered for inclusion. Reviews, case reports, and conference abstracts were excluded.

### 2.3 Study Selection and Data Extraction

Two independent reviewers screened the titles and abstracts of identified studies for eligibility. Any disagreements were resolved through discussion or by consulting a third reviewer. Full‐text articles were retrieved for further assessment if the studies met the eligibility criteria. Data from completed studies were extracted using a standardized data extraction form, including DOI, author, year, title, study type, inclusion criteria, intervention(s), participants' characteristics, key findings, and layman's findings. Data from trial registry records were extracted using a similar form, including NCT number, title, status, conditions, interventions, outcome measures, collaborators, age, phases, enrollment, study type, study designs, locations, and completion date.

### 2.4 Data Synthesis and Analysis

Due to the heterogeneity of healthcare approaches, outcome measures, and study populations across the included studies, a meta‐analysis was not deemed appropriate for this systematic review. Instead, a narrative synthesis approach was employed to discuss the findings, focusing on the implications for clinical practice and future research. This approach allowed for a comprehensive exploration of the various healthcare approaches for AS and their associated outcomes, while accounting for the complexity and diversity of the evidence. The narrative synthesis provided an overview of the evidence on different management approaches, as well as insights into potential avenues for future research. This approach also facilitated the identification of trends and patterns in the evidence, which were used to inform recommendations for clinical practice and future studies.

### 2.5 Risk of Bias Assessment

The risk of bias for the included RCTs was assessed using the revised Cochrane risk‐of‐bias tool for randomized trials (ROB 2) [18]. This tool evaluates the risk of bias across several domains, including randomization, deviations from intended interventions, missing outcome data, measurement of outcomes, and selective reporting. The studies were classified as low risk, high risk, or some concerns for each domain. The overall risk of bias for each study was then determined based on the most severe risk of bias level present in any domain.

In our study, the Newcastle–Ottawa Scale (NOS) tool was utilized to appraise the quality of observational studies included in our analysis [19]. This entailed a systematic evaluation of each study against the NOS's specified criteria across the categories of selection, comparability, and outcome or exposure. Studies were then awarded stars reflecting their adherence to these criteria, with the total possible score being 9 stars. This scoring facilitated a nuanced understanding of each study's methodological strengths and weaknesses, allowing for a more critical and informed synthesis of the evidence.

Two independent reviewers conducted the risk of bias assessment with any disagreements between the two reviewers were resolved through discussion or consultation with a third reviewer. The results of the risk of bias assessment were reported for each included study and considered when interpreting the findings of the systematic review.

## 3 Results

### 3.1 Study Selection

**Figure 1:**
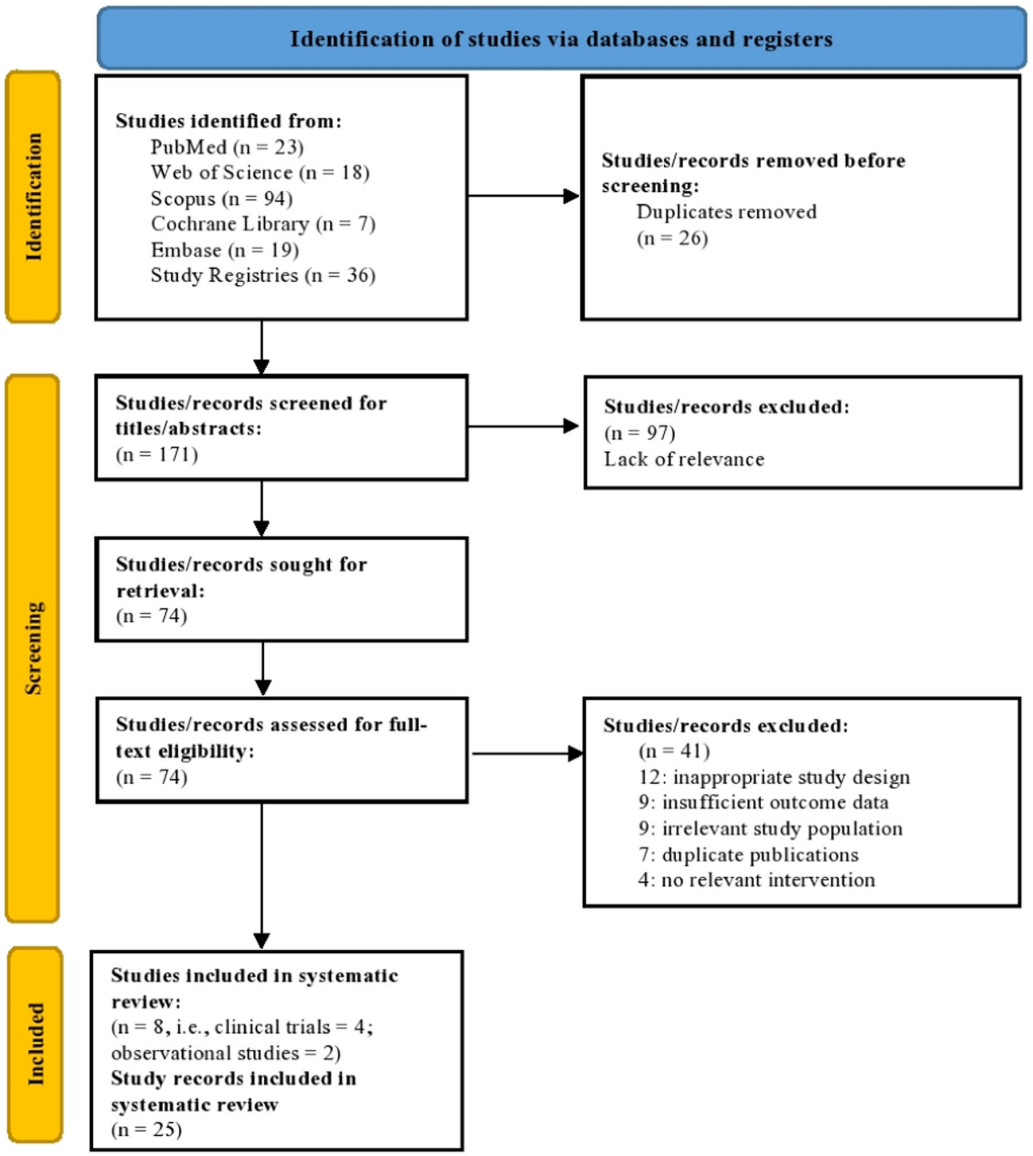
PRISMA flowchart depicting the study selection process.

A total of 197 studies were identified through the database search and trial registries. After removing 26 duplicates, 171 (86.8%) studies remained. These studies were then screened, and 74 (37.6%) were deemed relevant for retrieval and full‐text review. Following the full‐text review, 8 (4%) studies were included in the systematic review, with four original, completed clinical trials reported, and two observational studies. In addition, 25 ongoing study records were also included in the systematic review. The PRISMA flowchart is depicted in Figure 1.

### 3.2 Randomized Controlled Trials of AS

**Figure 2:**
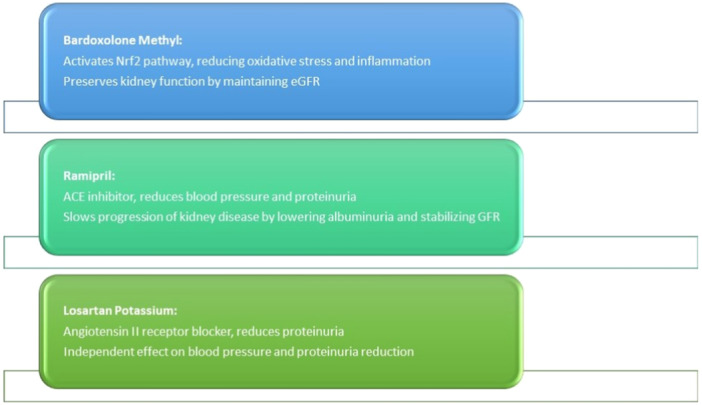
Mechanisms of action of the interventions.

**Figure 3:**
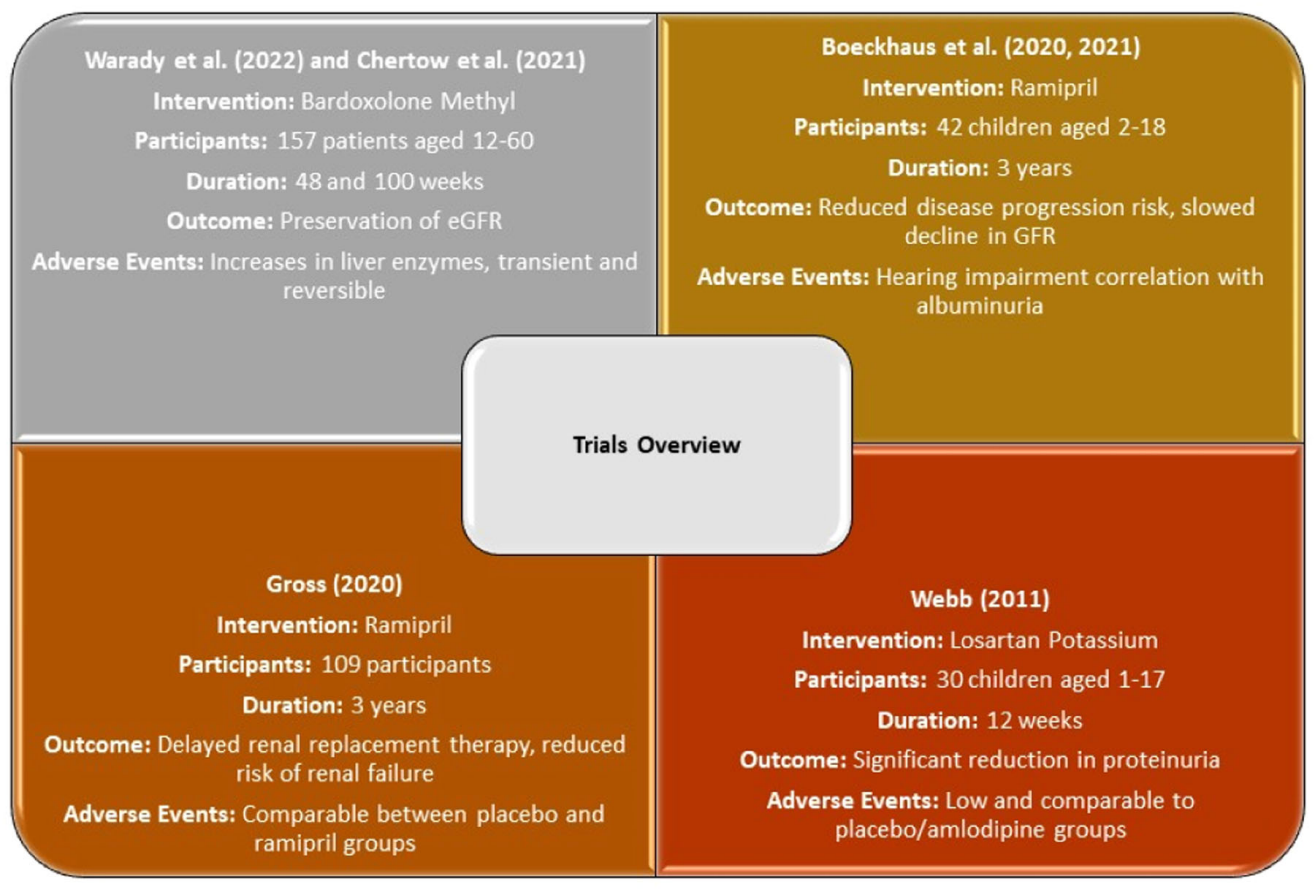
Overview of the included trials.

Four completed clinical trials were included in this systematic review. The mechanism of action of the interventions are presented in Figure 2. The characteristics are presented in Table 2. Figure 3 depicts an overview of the included trials.

**Table 2:** Characteristics of the included trials.

Author, Year	Title	Study type	Inclusion criteria	Intervention(s)	Participants' characteristics	Key findings	Layman finding
Warady et al. [20]	Effects of Bardoxolone Methyl in Alport Syndrome	Phase 2 (open‐label); Phase 3 (double‐blind, randomized, placebo‐controlled)	‐Male and female patients aged 12‐60 ‐Diagnosis of Alport syndrome by genetic testing or histologic assessment ‐Screening eGFR ≥ 30 and ≤ 90 mL/min/1.73 m2, with ≤ 25% difference between two values ‐ACR ≤ 3500 mg/g at Screen B visit ‐Stable ACE inhibitor/ARB use for 6 weeks before Screen A, or discontinued 8 weeks prior if medically contraindicated	Bardoxolone Methyl (RTA 402), dose escalated from 5 mg to max 20 or 30 mg depending on baseline proteinuria compared to placebo	‐Mean age: 39.2 years, 85% aged 18 or older, 58% female, 75% Caucasian ‐Mean age at Alport syndrome diagnosis: 29.7 years ‐20% histological diagnosis, 93% genetic diagnosis ‐62% X‐linked genetic subtype, 31% non‐X‐linked subtype	‐371 patients screened, 157 randomized (bardoxolone methyl: 77; placebo: 80) ‐15% aged < 18 years, 93% with confirmed genetic diagnosis ‐62% with X‐linked inheritance, mean baseline eGFR: 62.7 mL/min/1.73 m2, geometric mean UACR: 141.0 mg/g ‐Average annual eGFR decline: −4.9 mL/min/1.73 m2, 78% receiving ACE inhibitor/ARB therapy	Bardoxolone methyl treatment preserved eGFR in adolescent and adult Alport syndrome patients compared to placebo after 2 years; off‐treatment results with all available data showed no significant difference
Chertow et al. [21]	Study Design and Baseline Characteristics of the CARDINAL Trial: A Phase 3 Study of Bardoxolone Methyl in Patients with Alport Syndrome						
Boeckhaus et al. [22]	Precise variant interpretation, phenotype ascertainment, and genotype‐phenotype correlation of children in the EARLY PRO‐TECT Alport trial	Randomized, placebo‐controlled trial	‐Definitive diagnosis of Alport syndrome through kidney biopsy or mutation analysis and assessment of clinical criteria (hematuria, family history, ocular changes, labyrinthine hearing loss) ‐Alport syndrome levels 0, I, or II at screening (microhematuria, microalbuminuria, or proteinuria with GFR > 80 mL/min) ‐Patients aged between ≥ 24 months and < 18 years at screening	Oral treatment with 1 to 6 mg per body surface area ramipril once daily for 3 years compared to placebo	‐2 female and 49 male patients with a mean age of 9.0 ± 4.2 years at baseline ‐18 patients in AS stage 0, 23 patients in Stage I, and 10 patients in Stage II ‐82% X‐linked inheritance (42/51), 16% autosomal (8/51), and unknown in one patient (2%) ‐Median albuminuria at baseline: 61 mg albumin/gCrea (IQR 227.4 mg albumin/gCrea) ‐18 of 51 patients reported relatives with hearing loss (35%)	‐10–13‐year‐olds: 4PTA: 4.8 dB (healthy, n = 12), 41.4 dB (hearing impaired, *n* = 6.33%) ‐14–20‐year‐olds: 4PTA: 7.0 dB (healthy, *n* = 9), 48.2 dB (hearing impaired, *n* = 3.25%) ‐Hearing thresholds in hearing impaired group increased, particularly at 1–3 kHz frequencies ‐18% of children developed hearing loss, with maximum loss at 1–3 kHz	Hearing impairment in children with AS increased from 10% at baseline to 18% at the end of the trial
Boeckhaus et al. [23]	Characterization of Sensorineural Hearing Loss in Children with Alport Syndrome						
Gross et al. [24]	A multicenter, randomized, placebo‐controlled, double‐blind phase 3 trial with open‐arm comparison indicates safety and efficacy of nephroprotective therapy with ramipril in children with Alport's syndrome	Randomized, placebo‐controlled, double‐blind trial	‐Definitive diagnosis of Alport syndrome through kidney biopsy, mutation analysis, and clinical criteria assessment ‐Alport syndrome levels 0, I, or II at screening ‐Patients aged between ≥ 24 months and < 18 years	Ramipril treatment (1–6 mg daily for 3 years or until disease progression) compared to placebo	‐Mean age: 8.8 ± 4.2 years; 29% below 6 years of age ‐All patients had normal blood pressure, eGFR, few comorbidities, and few comedications ‐50% of patients had relatives with Alport syndrome who developed ESRF	‐Ramipril decreased risk of disease progression by almost half (hazard ratio 0.51) ‐Ramipril diminished albuminuria progression and decline in glomerular filtration ‐Adjusted analysis supported efficacy indications, with ramipril reducing progression by almost half (0.53) ‐Bayesian evidence synthesis resulted in a more precise estimate of hazard‐ratio (0.52)	Early initiation of therapy is safe and may slow renal failure by many years
Webb et al. [25]	Efficacy and safety of losartan in children with Alport syndrome‐‐results from a subgroup analysis of a prospective, randomized, placebo‐ or amlodipine‐controlled trial	Randomized, double‐blind, placebo‐controlled trial	‐Participants: 1 to 17 years of age ‐Able to provide first‐morning urine sample daily during the study ‐Documented history of proteinuria associated with chronic kidney disease of any origin	Treatments: Losartan potassium (tablets or liquid suspension), Amlodipine besylate (liquid suspension), or placebo for 12 weeks0	Six out of 30 children were hypertensive 15 children received losartan, 15 received amlodipine (4) or placebo (11)	‐Losartan significantly reduced proteinuria after 12 weeks compared to placebo/amlodipine (31.6% reduction vs. 2.3% increase, *p* = 0.01) ‐Adverse event incidence was low and comparable between losartan and placebo/amlodipine groups	Losartan was well tolerated and effective in children aged 1–17 years with proteinuria secondary to Alport syndrome, with or without hypertension

Abbreviations: 4PTA, 4‐frequency pure‐tone average; ACE, angiotensin‐converting enzyme; ACR, albumin‐to‐creatinine ratio; ARB, angiotensin II receptor blocker; AS, Alport Syndrome; dB, decibels; eGFR, estimated glomerular filtration rate; ESRF, end‐stage renal failure; gCrea, grams of creatinine; GFR, glomerular filtration rate; IQR, interquartile range; RTA 402, chemical identifier of Bardoxolone Methyl.

In a double‐blind, placebo‐controlled, randomized controlled trial (RCT) conducted by Warady et al. and Chertow et al., the effects of Bardoxolone Methyl on AS patients were rigorously evaluated. This study included 157 patients aged 12–60 across the United States, Europe, Japan, and Australia, who were diagnosed with AS and had specific estimated glomerular filtration rate (eGFR) and urinary albumin‐creatinine ratio (UACR) values. Bardoxolone Methyl was administered in doses ranging from 5 mg to 20 or 30 mg, depending on baseline proteinuria levels. The baseline characteristics of the patients were well‐balanced between the treatment groups. The mean baseline eGFR was 63 mL/min per 1.73 m² for both the bardoxolone methyl and placebo groups, indicating a moderate stage of kidney disease. The geometric mean baseline UACR was 148 mg/g in the bardoxolone methyl group and 134 mg/g in the placebo group. Additionally, 81% of patients in the bardoxolone methyl group and 75% in the placebo group were receiving an ACE inhibitor or ARB at the maximum tolerated dose. Patients receiving bardoxolone methyl experienced a notable preservation of eGFR relative to the placebo group at both 48 weeks (mean difference of 9.2 mL/min per 1.73 m²; 97.5% CI, 5.1 to 13.4 mL/min per 1.73 m²; *p* < 0.001) and 100 weeks (mean difference of 7.4 mL/min per 1.73 m²; 95% CI, 3.1 to 11.7 mL/min per 1.73 m²; *p* < 0.001). This effect was retained even after a 4‐week off‐treatment period, although the magnitude of the difference decreased (mean difference of 5.4 mL/min per 1.73 m² at 52 weeks; 97.5% CI, 1.8 to 9.1 mL/min per 1.73 m²; *p* < 0.001, and 4.4 mL/min per 1.73 m² at 104 weeks; 95% CI, 0.7 to 8.1 mL/min per 1.73 m²; *p* = 0.02). However, in post hoc analyses, the off‐treatment differences did not achieve statistical significance (mean difference of 1.5 mL/min per 1.73 m²; 95% CI, −1.9 to 4.9 mL/min per 1.73 m²; *p* = 0.38), highlighting a potential limitation in the durability of bardoxolone methyl's effects. The safety profile of bardoxolone methyl was another critical aspect of the study. Serious adverse events occurred in 10% of patients in the bardoxolone methyl group compared to 19% in the placebo group. Mild to moderate adverse events, particularly increases in liver enzymes, were significantly more common in the bardoxolone methyl group (alanine aminotransferase increase: 47% vs. 3%; aspartate aminotransferase increase: 25% vs. 1%). These increases were transient and reversible but highlight the need for careful monitoring. The effects of bardoxolone methyl in the adolescent subpopulation were particularly noteworthy. Adolescents treated with bardoxolone methyl showed a significant difference in eGFR preservation compared to the placebo group at 100 weeks (mean difference of 13.9 mL/min per 1.73 m²; 95% CI, 2.5 to 25.3 mL/min per 1.73 m²; *p* = 0.02) and 104 weeks (mean difference of 14.5 mL/min per 1.73 m²; 95% CI, 4.7 to 24.3 mL/min per 1.73 m²; *p* = 0.004). However, given the smaller sample size (23 adolescents), these results should be interpreted with caution [20, 21].

In the RCT conducted by Boeckhaus et al., the efficacy of oral ramipril, dosed at 1 to 6 mg per body surface area once daily for 3 years, was compared to placebo in patients with AS. The study included 42 children (40 males and 2 females) aged between 24 months and 18 years, all with a definitive diagnosis of AS. Out of the 66 children initially entered into the treatment phase, 60 were included in the final analysis after excluding five due to protocol violations or withdrawn consent. Molecular genetic testing was performed on 98% of the patients, revealing 53 different variants across the COL4A3, COL4A4, and COL4A5 genes. A significant proportion (58%) of these variants had not been previously described in the literature. According to the American College of Medical Genetics (ACMG) criteria, 87% of the cases were classified as “solved,” with likely pathogenic or pathogenic variants identified. At the end of the trial, hearing impairment in children increased from 10% at baseline to 18% in both treatment groups. Hearing loss was more prevalent among patients with severe genetic variants. For instance, half of the hearing‐impaired children with X‐linked AS (XLAS) had severe variants, while the other half had intermediate variants. The trial reported that the median albuminuria at baseline was 61 mg albumin/gCrea (IQR 227.4). Patients with hearing loss had a significantly higher median albuminuria of 300 mg albumin/gCrea compared to 45.8 mg albumin/gCrea in those with normal hearing (*p* ≤ 0.05). This suggests a correlation between higher albuminuria levels and the presence of hearing impairment. In the randomized arm, ramipril decreased the risk of disease progression by nearly half, with a hazard ratio of 0.51 (95% CI: 0.12–2.20). However, due to the small sample size, this reduction was not statistically significant. The ramipril group showed a diminished slope in the progression of albuminuria and a slower decline in glomerular filtration rate (GFR), though detailed p‐values for these outcomes were not reported [22, 23].

Gross conducted an RCT to evaluate the efficacy and safety of ramipril in patients with AS. The authors aimed to address the limited therapeutic options for AS and offers insight into the potential benefits of early intervention with ACE inhibitors. The trial enrolled a total of 109 participants, predominantly male (98.2%), with a small female representation (1.8%). Participants were divided into three treatment groups based on the timing of ACE inhibitor initiation: T‐I, T‐II, and T‐III. The primary endpoints included the delay in renal replacement therapy (RRT) and the progression of renal function decline. In the T‐I group, participants began ACE inhibitor therapy at a median age of 8 years, with an average treatment duration of 4 years. The early initiation of ramipril therapy significantly reduced the risk of disease progression, with a nearly 50% decrease in the risk of renal failure (HR 0.51, 95% CI 0.12–2.20, *p* = 0.03). For the T‐II group, ACE inhibitor therapy was initiated at a median age of 13 years, and the median age at commencement of RRT was delayed to 40 years. This group demonstrated a substantial delay in the need for RRT by approximately 27 years compared to untreated individuals, underscoring the high efficacy of early ramipril intervention. The T‐III group, which began ACE inhibitor therapy at a median age of 20 years, saw a delay in the progression to RRT by 3 years, with a median age at commencement of RRT of 25 years. The safety profile of ramipril was carefully monitored, with a total treatment duration amounting to 216.4 patient‐years. The incidence of adverse events (AEs) was comparable between the placebo and ramipril groups (0.63 vs. 0.60 events per patient‐year, respectively; rate ratio 0.96, 95% CI 0.63–1.45). Serious adverse events were rare and predominantly nondrug‐related, with no reported malignancies or deaths. Secondary and exploratory endpoints further supported the efficacy of ramipril. The progression of albuminuria was significantly lower in the ramipril group compared to the placebo (*p* < 0.05). Additionally, the loss of estimated glomerular filtration rate (eGFR) over the trial period was significantly lower in the ramipril group. The placebo group experienced a median eGFR loss of 9.8 mL/min over 3 years, whereas the ramipril group showed no significant eGFR decline (*p* = 0.01) [24].

The RCT conducted by Webb in 2011 focused on children aged 1 to 17 years with AS and proteinuria, with or without hypertension. A total of 30 children participated in the study, with six being hypertensive. The participants were randomly assigned to receive either Losartan potassium (*n* = 15), Amlodipine besylate (*n* = 4), or a placebo (*n* = 11). The gender distribution included 70% males (*n* = 21) and 30% females (*n* = 9). Baseline characteristics such as age, weight, height, BMI, ethnicity, pubertal status, duration of hypertension, and prior ACE‐I/ARB use were comparable across the treatment arms. The mean age of participants was 11.3 years (SD 4.7) in the Losartan group and 11.2 years (SD 3.4) in the placebo/amlodipine group. The mean weight was 41.8 kg (SD 18.9) for Losartan and 49.3 kg (SD 20.4) for placebo/amlodipine. The mean height was 141.8 cm (SD 23.2) for Losartan and 147.8 cm (SD 23.2) for placebo/amlodipine. The mean BMI was 19.7 kg/m² (SD 5.0) for Losartan and 21.6 kg/m² (SD 4.9) for placebo/amlodipine. After 12 weeks of treatment, Losartan significantly reduced proteinuria compared to placebo/amlodipine. The median change from baseline in the protein/creatinine (Pr/Cr) ratio was −14.7 mg/mmol (IQR: −49.7 to −5.7) for the Losartan group, while the placebo/amlodipine group showed a median change of 2.3 mg/mmol (IQR: −26.0 to 18.1). This difference was statistically significant (*p* = 0.018). The mixed model analysis further supported these findings, showing a percent reduction in proteinuria of 31.6% for Losartan (95% CI: 12.1 to 46.8) compared to −2.32% for placebo/amlodipine (95% CI: −28.8 to 18.8) (*p* = 0.010). Regarding blood pressure (BP) effects, the study reported a mean decrease in systolic blood pressure (SBP) of 1.9 mmHg (SD 11.0) in the Losartan group, compared to a mean increase of 2.9 mmHg (SD 6.6) in the placebo/amlodipine group, with a near‐significant difference (*p* = 0.051). For diastolic blood pressure (DBP), there was a mean increase of 3.9 mmHg (SD 8.4) in the Losartan group, and a mean increase of 3.5 mmHg (SD 11.1) in the placebo/amlodipine group (*p* = 0.82). Notably, there was no correlation between the magnitude of BP reduction and proteinuria reduction, indicating that the proteinuria‐lowering effect of Losartan was independent of its impact on BP. In terms of safety and tolerability, no deaths occurred during the study, and no patients discontinued treatment due to adverse events (AEs). AEs were reported in 18 patients (9 in the Losartan group and 9 in the placebo/amlodipine group), with only one AE in the Losartan group being drug‐related. There was one serious AE in the placebo/amlodipine group, which was not drug‐related. Predefined AEs of interest, such as angioedema, hyperkalemia, renal dysfunction, and hypotension, were not observed in either treatment arm. Plasma creatinine and potassium levels remained stable throughout the study [25].

### 3.3 Observational Studies of AS

The characteristics of the included observational studies are depicted in Table 3.

**Table 3:** Characteristics of the included observational studies and NOS findings.

Author, Year	Title	Study type	Inclusion criteria	Intervention(s)	Participants' characteristics	Key findings	Layman finding	NOS assessment
Gross et al. [26]	Early angiotensin‐converting enzyme inhibition in Alport syndrome delays renal failure and improves life expectancy	Observational, Longitudinal	Patients diagnosed with AS, including patients with varying stages of renal function at the initiation of therapy (hematuria/microalbuminuria, proteinuria, impaired renal function) and untreated relatives	Treatment with angiotensin‐converting enzyme inhibitors	Patients were categorized by renal function at the initiation of therapy: 33 with hematuria or microalbuminuria, 115 with proteinuria, 26 with impaired renal function, and 109 untreated relatives	‐Treatment with ACE inhibitors significantly delayed the start of dialysis, especially in those diagnosed with proteinuria and impaired renal function ‐Patients with hematuria or microalbuminuria did not advance to renal failure ‐Life expectancy also improved significantly in the treated cohort beyond the median age of 55 years of the no‐treatment cohort	The use of a specific medication (angiotensin‐converting enzyme inhibitors) in people with AS significantly delayed the need for dialysis and improved their life expectancy. This was especially true when treatment was started earlier in the disease's progression	Selection: 3 Comparability: 2 Outcome/Exposure: 3 Total Score: 8
Jais et al. [27]	X‐linked Alport Syndrome: Natural History in 195 Families and Genotype‐ Phenotype Correlations in Males	Observational Study	‐Patients diagnosed with AS ‐Male patients with COL4A5 mutation	Not Applicable	‐Total number of families: 329 ‐Families with X‐linked transmission: 250 ‐Male patients with COL4A5 mutation: 401‐All male patients were hematuric	‐The rate of progression to end‐stage renal failure and deafness was mutation‐dependent ‐Large deletions, nonsense mutations, or small mutations changing the reading frame conferred a 90% probability of developing end‐stage renal failure before 30 years of age ‐Patients with missense or splice site mutations had a 50% and 70% risk, respectively, of developing end‐stage renal failure before 30 years of age ‐The risk of developing hearing loss before 30 years of age was approximately 60% for patients with missense mutations, compared to 90% for other types of mutations ‐The natural history of X‐linked AS and correlations with COL4A5 mutations have been established in a large cohort of male patients	The study involved 329 families, specifically focusing on male patients with a COL4A5 gene mutation, found that the risk of developing severe kidney failure and hearing loss varied depending on the type of mutation	Selection: 3 Comparability: 1 Outcome/Exposure: 2 Total Score: 6

**Abbreviations:** AS, Alport syndrome; ACE, angiotensin‐converting enzyme.

Gross conducted an observational, longitudinal study focusing on patients diagnosed with AS at various stages of renal function. The patient groups included those with hematuria or microalbuminuria, those with proteinuria, those with impaired renal function, and a group of untreated relatives. The intervention studied was the treatment with angiotensin‐converting enzyme inhibitors. Gross et al. found that this treatment significantly delayed the need for dialysis, especially in those initially diagnosed with proteinuria and impaired renal function. Those with hematuria or microalbuminuria did not advance to renal failure. The life expectancy improved notably in the treated cohort, extending beyond the median age of 55 years observed in the no‐treatment group. The findings emphasized the crucial role of early diagnosis and treatment with ACE inhibitors in improving the prognosis for patients with AS [26].

Jais [27] published an observational study focused on male patients diagnosed with X‐linked AS and carrying the COL4A5 mutation. This study involved 329 families, with 250 exhibiting X‐linked transmission. There were 401 male patients with the COL4A5 mutation, all of whom were hematuric. Philippe discovered that the rate of progression to end‐stage renal failure and deafness was dependent on the type of mutation. Large deletions, nonsense mutations, or small mutations changing the reading frame conferred a 90% probability of developing end‐stage renal failure before 30 years of age. Those with missense or splice site mutations had a 50% and 70% risk, respectively, of developing end‐stage renal failure before reaching 30. As for hearing loss, the risk before 30 years of age was approximately 60% for patients with missense mutations, compared to 90% for other types of mutations. The study thus established the natural history of X‐linked AS and correlations with COL4A5 mutations in a large male patient cohort, highlighting that the risk of severe kidney failure and hearing loss varied depending on the mutation type [27].

### 3.4 Synthesis of Clinical Trial Registry Records

A comprehensive review of the ongoing clinical trials related to AS was conducted, including a total of 25 ongoing trial registry records. The status of these records comprised 10 completed, 10 currently recruiting, 2 not yet recruiting, and 3 terminated trials. The conditions under investigation included AS, X‐Linked AS, and various associated kidney diseases. Information on these records is enlisted in Table 4.

**Table 4:** Study record identifiers, title, status, interventions, outcomes and collaborators.

No.	NCT Number	Title	Status	Conditions	Interventions	Outcome measures	Collaborators
1	NCT03019185	A Phase 2/3 Trial of the Efficacy and Safety of Bardoxolone Methyl in Patients With Alport Syndrome ‐ CARDINAL	Completed	Alport Syndrome	Drug: Placebo Oral Capsule; Drug: Bardoxolone Methyl	Increase in eGFR from baseline; Increase in eGFR from baseline following a 4‐week drug treatment withdrawal period; Number of participants with treatment‐related adverse events as assessed by CTCAE v4.0	Reata Pharmaceuticals Inc.
2	NCT03373786	A Study of RG‐012 in Subjects With Alport Syndrome	Completed	Alport Syndrome	Drug: RG012	Safety—Adverse Events; Effect of RG‐012 on renal microRNA‐21 (miR‐21); Pharmacokinetic (PK) parameter— Cmax; Pharmacokinetic (PK) parameter—Tmax; Pharmacokinetic (PK) parameter— AUC	Genzyme, a Sanofi Company; Sanofi
3	NCT04937907	Study of Hydroxychloroquine in Patients With X‐linked Alport Syndrome in China (CHXLAS)	Recruiting	Alport Syndrome, X‐Linked	Drug: Hydroxychloroquine Sulfate 100 milligram (mg) Tab; Drug: Benazepril hydrochloride 10 milligram (mg) Tab	Change in urinary erythrocyte count(/HP); Change in 24‐h urinary protein quantity; Change in urinary albumin characterization; Change in urinary albumin to creatinine ratio; Change in urinary erythrocyte count(urinary sediment analyzer); Change in eGFR from baseline; Number of participants with treatment‐related adverse events	Shanghai Children's Hospital
4	NCT05448755	A Study of ELX‐02 in Patients With Alport Syndrome	Recruiting	Alport Syndrome	Drug: ELX‐02	The incidence and characteristics of adverse events; Change in proteinuria; Change in Col IV expression in renal biopsy; Change in hematuria	Eloxx Pharmaceuticals Inc.
5	NCT04947813	Genotype‐Phenotype Correlations in Patients With Alport Syndrome	Recruiting	Alport Syndrome	NR	Identification COL4A3/COL4A4/COL4A5 variants of Alport Syndrome; Identification genotype‐phenotype correlations of Alport Syndrome	Xinhua Hospital, Shanghai Jiao Tong University School of Medicine
6	NCT02855268	Study of Lademirsen (SAR339375) in Patients With Alport Syndrome (HERA)	Terminated	Alport's Syndrome	Drug: lademirsen (SAR339375); Drug: Placebo	Number of participants with adverse events; Annualized change in estimated glomerular filtration rate eGFR from baseline; Pharmacokinetics (PK): Maximum concentration in plasma (Cmax); Pharmacokinetics (PK): Trough plasma concentration (Ctrough); Number of participants with anti‐drug antibodies (ADAs); Number of participant with adverse events associated to ADAs; Percent change in eGFR values; Proportion of subjects who reach end staged renal disease (ESRD); Proportion of subjects with a reduction from baseline in eGFR; Change in circulating miR‐21; Change in blood urea nitrogen; Change in protein/creatinine ratio and albumin/creatinine ratio in urine only; Change in epidermal growth factor (EGF) in urine only; Change in creatinine in both blood and urine; Change in Cystatin C in both blood and urine; Change in transforming growth factor‐Î² (TGF‐Î²) in both blood and urine; Change in neutrophil gelatinase‐associated lipocalin (NGAL) in both blood and urine	Genzyme, a Sanofi Company; Sanofi
7	NCT05133050	Safety and Efficacy of ACEI in Alport Syndrome Patients With COL4A3/COL4A4/COL4A5 Variants	Not yet recruiting	Alport Syndrome	Drug: Ramipril	Disease progression time; 5‐year disease progression rate and eGFR slope	Xinhua Hospital, Shanghai Jiao Tong University School of Medicine
8	NCT00481130	Alport Syndrome Treatments and Outcomes Registry (ASTOR)	Recruiting	Alport Syndrome	NR	Data Collection: natural history study	University of Minnesota
9	NCT01696253	Multi‐center Controlled Clinical Trials in Alport Syndrome‐A Feasibility Study	Completed	Alport Syndrome	NR	Multi‐center Controlled Clinical Trials in Alport Syndrome‐A Feasibility Study	University of Minnesota; University of Utah; Peking University First Hospital; University of Toronto; University of GÃ¶ttingen; HÃ´pital Necker‐Enfants Malades
10	NCT02136862	ATHENA: Natural History of Disease Study in Alport Syndrome Patients (RG012‐01)	Completed	Alport Syndrome Patients With eGFR Between 45–90 mL/Min/1.73;m^2^	NR	To characterize the natural decline of renal function markers (Glomerular Filtration Rate [GFR] and creatinine) in patients with Alport syndrome over the course of up to 120 weeks	Genzyme, a Sanofi Company; Sanofi
11	NCT00309257	Effects of an Intensified Treatment With ACE‐I, ATA II and Statins in Alport Syndrome	Completed	Alport Syndrome	Drug: ACE I, ATA II and Statins; Drug: Benazepril, Valsartan and Fluvastatin	Urinary protein excretion; Blood pressure; Urinary podocyte excretion	Mario Negri Institute for Pharmacological Research
12	NCT01602835	Human Urine Sample Collection for Alport Nephropathy Biomarker Studies	Terminated	Alport Syndrome	NR	NR	Novartis Pharmaceuticals; Novartis
13	NCT05655728	Treatment With Metformin in Chinese Children With Alport Syndrome	Not yet recruiting	Alport Syndrome; Metformin	Drug: Metformin; Other: Placebo	Efficacy of metformin for Alport syndrome; Safety of metformin for Alport syndrome	Peking University First Hospital
14	NCT01705132	Urinary Biomarkers of the Progression of Alport Kidney Disease	Completed	Alport Syndrome	NR	Urine levels of biomarkers, corrected for urine creatinine, in Alport subjects stratified by magnitude of proteinura.	University of Minnesota; Novartis
15	NCT02718027	Biomarker for Alport Syndrome (BioAlport)	Terminated	Nephritis, Hereditary; Hematuria‐Nephropathy‐Deafness Syndrome	NR	Identification of Alport Syndrome biomarker/s; Exploring the clinical robustness, specificity, and long‐term variability of Alport syndrome biomarker/s	CENTOGENE GmbH Rostock
16	NCT00622544	A Prospective Study of Microalbuminuria in Untreated Boys With Alport Syndrome (MA)	Completed	Alport Syndrome; Kidney Disease	NR	Number of Subjects Developing Microalbuminuria During Study Period; Number of Subjects Developing Proteinuria During the Study Period	University of Minnesota; University of Utah
17	NCT03074357	Urine, DNA and Clinical Information Collection From Patients With Alport Nephropathy.	Completed	Alport Nephropathy	NR	Urine levels of biomarkers ‐ corrected for urine creatinine; Urine level of biomarkers ‐ corrected for urine creatinine	Novartis Pharmaceuticals; Novartis
18	NCT05267262	Study to Evaluate R3R01 in Patients With Alport Syndrome and Patients With Focal Segmental Glomerulosclerosis	Recruiting	Alport Syndrome; Focal Segmental Glomerulosclerosis	Drug: R3R01	Incidence of adverse events (Safety and Tolerability); Assess change in urine creatinine protein ratio; Change in quality‐of‐life assessment from baseline to end of treatment and to the end of the follow‐up period by cohort for adults; Change in quality‐of‐life assessment from baseline to end of treatment and to the end of the follow‐up period by cohort for children	River 3 Renal Corp.
19	NCT02378805	European Alport Therapy Registry ‐ European Initiative Towards Delaying Renal Failure in Alport Syndrome	Recruiting	Alport Syndrome; Hereditary Kidney Disease; Pediatric Kidney Disease; Thin Basement Membrane Disease; Familial Benign Hematuria	Drug: ACE‐inhibitor; Drug: AT1‐inhibitor; Drug: HMG‐Coenzyme inhibitor (statin); Drug: Spironolactone; Drug: Paricalcitol; Drug: SGLT2 inhibitor	End stage renal disease; life‐expectancy; proteinuria after initiation of ACE‐inhibitor‐therapy; proportion of patients with a clinical diagnosis of hypertension; proportion of patients experiencing side effects from ACE‐inhibitors	University Hospital Goettingen; Society for Pediatric Nephrology (Germany); Deutsche Gesellschaft fÃ¼r Nephrologie; Alport Selbsthilfe e.V.; Association pour l'Information et la Recherche sur les Maladies RÃ©nales GÃ©nÃ©tiques (AIRG); KfH Foundation Preventive Medicine
20	NCT01485978	Efficacy and Safety Study to Delay Renal Failure in Children With Alport Syndrome	Completed	Renal Insufficiency, Chronic	Drug: Ramipril; Drug: placebo to ramipril	Time to next disease level; Incidence of Adverse Drug Events before progression; Albuminuria after three years; Adverse Drug Events over three years	Institut fuer anwendungsorientierte Forschung und klinische Studien GmbH; University Medical Center Goettingen; German Federal Ministry of Education and Research
21	NCT03749447	An Extended Access Program for Bardoxolone Methyl in Patients With CKD (EAGLE)	Recruiting	Chronic Kidney Diseases; Alport Syndrome; Autosomal Dominant Polycystic Kidney	Drug: Bardoxolone methyl	Long‐term safety: by incidence of adverse events and serious adverse events	Reata Pharmaceuticals Inc.
22	NCT05003986	Study of Sparsentan Treatment in Pediatrics With Proteinuric Glomerular Diseases (EPPIK)	Recruiting	Focal Segmental Glomerulosclerosis; Minimal Change Disease; Immunoglobulin A Nephropathy; IgA Vasculitis; Alport Syndrome	Drug: Sparsentan	Incidence of treatment‐emergent adverse events (TEAEs), serious adverse events (SAEs), AEs leading to treatment discontinuation, and adverse events of interest (AEOIs); Urine protein/creatinine ratio (UP/C) at week 108; Observed plasma Pharmacokinetic (PK) concentrations; Steady‐state PK parameters area under the plasma concentration‐time curve during a dosing interval ([AUCÏ„]); Steady‐state PK parameters [Cmax_ss]; Steady‐state PK parameters [Cmin_ss]; Urine albumin/creatinine ratio (UA/C) over the 112 weeks; Urine protein/creatinine ratio (UP/C) over the 112 weeks; Estimated glomerular filtration rate (eGFR) over the 112 weeks; Proportion of subjects with FSGS and/or MCD histological patterns achieving partial remission	Travere Therapeutics Inc.
23	NCT01465126	Enalapril in Collagen Type 4 Nephropathy	Completed	Collagen Type‐4 Nephropathies	NR	Proteinuria; evaluate the creatinine clearance during treatment with enalapril	University of Sao Paulo
24	NCT04573920	Atrasentan in Patients With Proteinuric Glomerular Diseases (AFFINITY)	Recruiting	IgA Nephropathy; Focal Segmental Glomerulosclerosis; Alport Syndrome; Diabetic Kidney Disease; Diabetic Nephropathy Type 2; Immunoglobulin A Nephropathy	Drug: Atrasentan	Change in proteinuria for IgAN, FSGS, and Alport syndrome patients receiving 0.75 mg atrasentan QD; Change in albuminuria for DKD patients; Change in proteinuria for FSGS patients at 1.5 mg dose	Chinook Therapeutics U.S. Inc.; Chinook Therapeutics Inc.
25	NCT05687474	Baby Detect: Genomic Newborn Screening	Recruiting	Alport Syndrome and others	NR	Acceptability; Feasability ‐ timing; Feasabilty ‐ reliability; Consequence of NBS on early treatment access ‐ timing; Consequence of NBS on early treatment access ‐ frequency; To improve the detection technique for disease related mutations that are not detected in classical screening by improving the classification of unspecified variants.	Laurent Servais; Centre Hospitalier Universitaire de Liege; University of Liege; Centre Hospitalier RÃ©gional de la Citadelle

Eleven (44%) of the 25 clinical trials administered therapeutic interventions, while the remaining studies do not involve specific interventions, as they focus on natural history, data collection, biomarker identification, and feasibility assessments. The interventions are enlisted as follows:

1. Antioxidant and anti‐inflammatory agents: NCT03019185: Bardoxolone Methyl.
2. Anti‐fibrotic agents targeting microRNA‐21: NCT03373786: RG012 and NCT02855268: Lademirsen (SAR339375).
3. Immunomodulatory agents: NCT04937907: Hydroxychloroquine Sulfate.
4. Protein translation modulators: NCT05448755: ELX‐02.
5. Renin‐angiotensin‐aldosterone system (RAAS) inhibitors: NCT05133050: Ramipril; NCT01705132: ACE I, ATA II, and Statins (Benazepril, Valsartan, Fluvastatin).
6. Metformin: NCT05655728: Metformin.
7. Renin receptor antagonist: NCT05003986: Sparsentan.
8. Endothelin receptor antagonist: NCT04573920: Atrasentan.
9. ACE inhibitor: NCT01465126: Enalapril.

The outcome measures for the clinical trials related to AS are organized thematically as follows:

1. Kidney function and proteinuria measures: increase in eGFR from baseline, change in proteinuria, and change in urinary albumin to creatinine ratio.
2. Safety and adverse events: number of participants with treatment‐related adverse events as assessed by CTCAE v4.0, incidence and characteristics of adverse events, the incidence of treatment‐emergent adverse events (TEAEs), serious adverse events (SAEs), AEs leading to treatment discontinuation, and adverse events of interest (AEOIs).
3. Pharmacokinetics: Pharmacokinetic (PK) parameters—Cmax, Tmax, and AUC.
4. Biomarkers and molecular measures: change in renal microRNA‐21 (miR‐21), change in Col IV expression in renal biopsy, identification of AS biomarker/s and genotype‐phenotype correlations, and change in circulating miR‐21 and other biomarkers (e.g., TGF‐β, NGAL).
5. QoL and patient‐reported outcomes: change in quality‐of‐life assessment from baseline to end of treatment and the end of the follow‐up period.
6. Disease progression and renal outcomes: time to next disease level, disease progression time and 5‐year disease progression rate, and proportion of subjects who reach ESRD.
7. Other outcome measures: change in blood pressure, annualized change in estimated glomerular filtration rate (eGFR) from baseline, change in urinary erythrocyte count, natural history data collection, the feasibility of multi‐center controlled clinical trials in AS, and acceptability and feasibility of newborn screening (NBS) and early treatment access.

Participants belonged to various age groups, as listed in Table 4. The trials included children as young as 1‐year‐old and adults up to 99 years old. Some trials focused on specific age ranges, such as 12–60, 18–65, or 3–18 years, while others encompassed broader categories, including children, adults, and older adults.

Out of the 25 clinical trials of AS, seven were Phase 2 trials, one was a Phase 1 trial, one was Phase 2/3, two were in Phase 3, and one was a Phase 4 trial. The remaining 13 trials were classified as “Not Applicable” due to their nature, such as observational or natural history studies, or not fitting into the conventional phase classification. A total of 52,135 participants were enrolled in the trials.

The clinical trials spanned multiple countries across the globe. The majority of these trials took place in the United States (*n* = 14; 56%). Other notable locations include the United Kingdom (*n* = 8, 32%), Germany (*n* = 7, 28%), Australia (*n* = 5, 20%), France (*n* = 5, 20%), and Spain (*n* = 5, 20%). Trials were additionally conducted in China, Japan, Belgium, Italy, the Netherlands, Canada, Puerto Rico, Albania, Georgia, India, Lithuania, Pakistan, Romania, Sri Lanka, and South Korea (Table 5).

**Table 5:** Sample traits, enrollment, study type and design, location, and completion date of study records.

No.	Age	Phases	Enrollment	Study type	Study designs	Locations	Completion date
1	12–60 Years	Phase 2; Phase 3	187	Interventional	Allocation: Randomized; Intervention Model: Parallel Assignment; Masking: Triple (Participant, Care Provider, Investigator); Primary Purpose: Treatment	United States; Australia; France; Germany; Japan; Puerto Rico; Spain; United Kingdom	30‐Oct‐20
2	18–65 Years	Phase 1	4	Interventional	Allocation: Non‐Randomized; Intervention Model: Sequential Assignment; Masking: Double (Participant, Investigator); Primary Purpose: Treatment	United States	20‐May‐19
3	3–18 Years	Phase 2	50	Interventional	Allocation: Randomized; Intervention Model: Parallel Assignment; Masking: None (Open Label); Primary Purpose: Treatment	China	30‐Jun‐23
4	6–30 Years	Phase 2	8	Interventional	Allocation: N/A; Intervention Model: Single Group Assignment; Masking: None (Open Label); Primary Purpose: Treatment	Australia; United Kingdom	30‐May‐23
5	Child, Adult, Older Adult	Not Applicable	8165	Observational	Observational Model: Cohort; Time Perspective: Prospective	China	31‐Dec‐30
6	18–55 Years	Phase 2	43	Interventional	Allocation: Randomized; Intervention Model: Parallel Assignment; Masking: Triple (Participant, Care Provider, Investigator); Primary Purpose: Treatment	United States; Australia; China; France; Germany; Spain; United Kingdom	22‐Sep‐22
7	30–50 Years	Not Applicable	510	Interventional	Allocation: Randomized; Intervention Model: Parallel Assignment; Masking: None (Open Label); Primary Purpose: Treatment	China	31‐Dec‐26
8	0–99 Years	Not Applicable	1000	Observational	Observational Model: Family‐Based; Time Perspective: Other	United States	Jan‐30
9	1 Year and Older		360	Observational	Observational Model: Case‐Only; Time Perspective: Other	United States	Dec‐17
10	12–65 Years	Not Applicable	165	Observational	Observational Model: Cohort; Time Perspective: Prospective	United States; Australia; Canada; Germany; United Kingdom	18‐Dec‐17
11	15–70 Years	Phase 2	9	Interventional	Allocation: N/A; Intervention Model: Single Group Assignment; Masking: None (Open Label); Primary Purpose: Treatment	Italy	Oct‐09
12	5 Years and Older	Not Applicable	80	Observational	Observational Model: Cohort; Time Perspective: Cross‐Sectional	Not Reported	May‐13
13	10–18 Years	Phase 4	78	Interventional	Allocation: Randomized; Intervention Model: Parallel Assignment; Masking: Quadruple (Participant, Care Provider, Investigator, Outcomes Assessor); Primary Purpose: Treatment	Not Reported	20‐Apr‐25
14	5–65 Years	Not Applicable	80	Observational	Observational Model: Family‐Based; Time Perspective: Prospective	United States	Jul‐13
15	2 Months‐50 Years	Not Applicable	12	Observational	Observational Model: Cohort; Time Perspective: Prospective	Albania, Georgia, India, Lithuania, Pakistan, Romania, Sri Lanka	31‐Dec‐22
16	Up To 18 Years	Not Applicable	44	Observational	Observational Model: Other; Time Perspective: Prospective	United States	Jul‐12
17	5 Years and Older	Not Applicable	68	Observational	Observational Model: Other; Time Perspective: Retrospective	United States	23‐Mar‐18
18	12 Years and Older	Phase 2	50	Interventional	Allocation: Non‐Randomized; Intervention Model: Single Group Assignment; Masking: None (Open Label); Primary Purpose: Treatment	United States; Belgium; France; Germany; Netherlands; United Kingdom	15‐Dec‐23
19	Child, Adult, Older Adult	Not Applicable	500	Observational	Observational Model: Cohort; Time Perspective: Cross‐Sectional	Germany	Jan‐35
20	24 Months‐18 Years	Phase 3	66	Interventional	Allocation: Randomized; Intervention Model: Parallel Assignment; Masking: Double (Participant, Investigator); Primary Purpose: Treatment	Germany	Mar‐19
21	12 Years and Older	Phase 3	480	Interventional	Allocation: N/A; Intervention Model: Single Group Assignment; Masking: None (Open Label); Primary Purpose: Treatment	United States; Australia; France; Japan; Puerto Rico; Spain	Dec‐25
22	1–17 Years	Phase 2	57	Interventional	Allocation: Non‐Randomized; Intervention Model: Parallel Assignment; Masking: None (Open Label); Primary Purpose: Treatment	United States, Germany, Italy, Netherlands, Poland, Spain, United Kingdom	1‐Jun‐25
23	Up To 18 Years	Not Applicable	19	Observational	Observational Model: Case‐Only; Time Perspective: Retrospective	Not Reported	Oct‐11
24	18 Years and Older	Phase 2	100	Interventional	Allocation: Non‐Randomized; Intervention Model: Single Group Assignment; Masking: None (Open Label); Primary Purpose: Treatment	United States, Australia, Italy, Korea (Republic of), Spain, England (United Kingdom)	1‐Feb‐26
25	Up To 28 Days	Not Applicable	40000	Observational	Observational Model: Other; Time Perspective: Prospective	Belgium	31‐Aug‐25

### 3.5 Risk of Bias Assessment Findings

The study conducted by Gross in 2012 was awarded a total NOS score of 8 out of a possible 9 points. This high score reflects the study's strong selection criteria, excellent comparability between cohorts, and comprehensive outcome assessment. Specifically, the study was recognized for its clear inclusion criteria, accounting for varying stages of renal function and the treatment impact on life expectancy and the delay in the start of dialysis among AS patients.

On the other hand, the study by Philippe in 2000 received a NOS score of 6 out of 9. This score indicates a solid study design with well‐defined diagnostic criteria and a large cohort, contributing to the understanding of genotype‐phenotype correlations in AS. However, the study scored slightly lower in comparability due to less detailed matching criteria. Despite this, it offered valuable insights into the mutation‐dependent progression of AS, highlighting the variable risk of developing end‐stage renal failure and deafness associated with different COL4A5 mutations.

**Figure 4:**
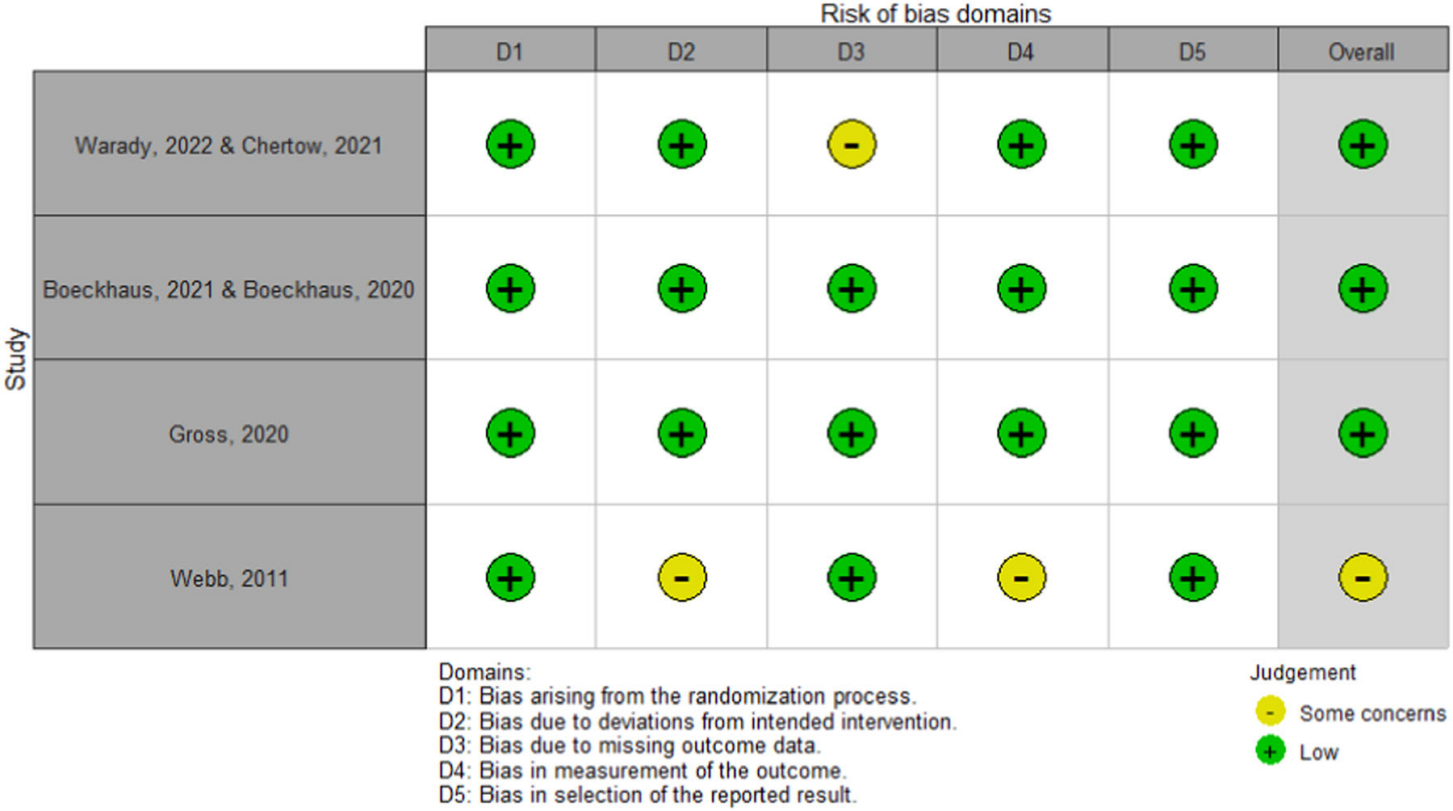
Traffic light plot representing the risk of bias across the included completed RCTs.

The risk of bias for the included studies was assessed using Cochrane's ROB 2 tool. For the trial by Warady (2022)/Chertow (2021), the risk of bias was assessed as low in D1, 2, 4, and 5, while some concerns were noted in Domain 3. Overall, the study was considered to have a low risk of bias. Boeckhaus/Boeckhaus were rated as low risk across all domains, resulting in an overall low risk of bias, though such assessments could potentially be downgraded in other studies due to factors like unblinding or high attrition rates. Gross also had a low risk of bias across all domains, indicating an overall low risk of bias. Lastly, Webb was assessed as low in Domains 1, 3, and 5, with some concerns identified in Domains 2 and 4. Consequently, the study was considered to have some concerns in terms of the risk of bias. The traffic light plot is illustrated in Figure 4.

## 4 Discussion

The findings of this systematic review highlight the potential benefits of various therapeutic interventions for patients with AS, including Bardoxolone Methyl, ramipril, and Losartan. While these findings are suggestive of benefits in the clinical trial setting, it is pertinent that these findings are interpreted with caution. These results suggest that early initiation of therapy could help preserve kidney function and potentially slow the progression of renal failure. However, the available evidence is limited, warranting further investigation in future research [28, 29]. Warady and Chertow demonstrated that Bardoxolone Methyl treatment preserved eGFR in adolescent and adult AS patients compared to the placebo group after 2 years. Findings reported by Boeckhaus, Boeckhaus, and Gross suggest that ramipril treatment could slow disease progression and reduce the risk of renal failure. Webb reported that Losartan effectively reduced proteinuria in children with AS. These treatments target various aspects of AS pathophysiology, including oxidative stress, inflammation, and proteinuria. However, our understanding of these interventions' long‐term effects and their applicability to a diverse range of patients remains limited [30].

Current guidelines in the United States for managing patients with AS who are in stages 1–4 of kidney disease (with an eGFR) between 15 and 90+ mL/min/1.73 m^2^) emphasize the early initiation of treatment with ACE inhibitors or ARBs [31]. This approach is particularly crucial for individuals with X‐linked or autosomal recessive forms of AS, for whom starting treatment as soon as possible after diagnosis and escalating to the highest tolerable dose is advised. The treatment strategy for X‐linked females and patients with autosomal dominant AS involves a more nuanced assessment, taking into account the presence of proteinuria or hypertension before initiating therapy. Evidence indicates that early use of RAAS inhibitors can significantly influence the trajectory of disease progression. Although ACE inhibitors are often the first choice due to their slightly superior efficacy and the abundance of supporting data, ARBs are frequently selected for their improved tolerability. In addition, sodium‐glucose cotransporter‐2 (SGLT‐2) inhibitors are being adopted in treatment regimens for their kidney‐protective effects, despite the lack of specific studies in AS patients, leveraging their established benefits in chronic kidney disease (CKD) patients without diabetes to reduce the risk of progressing to kidney failure.

### 4.1 Implications for Male Patients

Based on the clinical trials and observational studies evaluated in this systematic review, the implications for male patients diagnosed with AS are multifaceted.

Firstly, treatment with Bardoxolone Methyl has been shown to preserve eGFR in adolescent and adult male patients, demonstrating its potential effectiveness in slowing kidney function decline in male patients (Warady et al.). However, off‐treatment results indicated no significant difference, suggesting the necessity of consistent treatment.

Furthermore, studies indicate a potential therapeutic role of Ramipril in the treatment of AS in male patients. Ramipril treatment has been observed to safely delay renal failure progression and could be beneficial, particularly when initiated early in the disease course (Gross et al.).

In the study by Webb et al., the use of Losartan was found to significantly reduce proteinuria in children with AS, demonstrating its effectiveness and tolerance in this population. This suggests that Losartan could be a potential therapeutic option for male pediatric patients with proteinuria secondary to AS.

Looking at observational studies, Gross et al. found that the use of angiotensin‐converting enzyme inhibitors (ACE inhibitors) in male AS patients significantly delayed the onset of dialysis and improved their life expectancy. This indicates the potential value of early ACE inhibitor treatment in male patients with AS, particularly for those diagnosed with proteinuria and impaired renal function.

The study by Jais et al. [27] highlighted the genetic aspect of AS in males, focusing on those with the COL4A5 mutation linked to X‐linked AS. The rate of progression to end‐stage renal failure and deafness was found to be dependent on the type of mutation. Therefore, male patients with certain types of mutations, such as large deletions, nonsense mutations, or small mutations altering the reading frame, have a significantly increased risk of developing end‐stage renal failure before the age of 30. This finding underscores the critical need for early genetic testing and individualized management plans for male AS patients.

Male patients with AS may benefit from therapies such as Bardoxolone Methyl, Ramipril, and Losartan, as well as ACE inhibitors. These treatments generally have better benefits when initiated early in the disease course. However, in cases of X‐linked AS in males, the type of COL4A5 mutation greatly influences disease progression and the development of serious complications such as end‐stage renal failure and deafness, underlining the importance of early genetic testing and personalized care in this population.

### 4.2 Enhanced Endpoint Focus for Future AS Trials

Future clinical trials in AS should prioritize endpoints that bridge laboratory findings with tangible clinical benefits. We propose a robust assessment framework where enhancements in eGFR, insulin clearance, and reductions in albuminuria are directly correlated with the critical clinical endpoint of delaying the onset of dialysis. This targeted approach allows us to demonstrate the clinical utility of laboratory markers in predicting long‐term patient outcomes more effectively.

In addition to efficacy, a thorough evaluation of safety profiles and adverse events associated with therapeutic interventions is essential. Detailed reporting of these aspects will aid clinicians in making informed decisions and ensure that patient safety is prioritized, adhering to rigorous pharmacovigilance standards.

Furthermore, the genetic heterogeneity observed in AS necessitates the inclusion of genetic profiling in patient cohort definitions. By incorporating genetic markers such as variants in COL4A3, COL4A4, and COL4A5, our study design can address the variable progression rates from diagnosis to renal failure. This strategic focus will enhance the precision of intervention assessments, reducing potential biases and optimizing therapeutic outcomes for distinct genetic subgroups.

This refined focus on endpoints and patient selection criteria is designed to ensure that future trials not only address the mechanistic efficacy of treatments but also their real‐world applicability and safety, thereby enhancing the overall quality of evidence and supporting the development of more personalized treatment strategies for AS.

### 4.3 Strengths of This Systematic Review

Strengths of this study include a novel and current synthesis of completed clinical trials related to AS, providing a comprehensive literature overview of the interventions and outcomes assessed up until December 24, 2023. This study includes 25 study records pooling 52,135 participants conducted globally, which offers insights into the potential generalizability of the findings.

### 4.4 Limitations of the Systematic Review

There are several limitations to consider. Firstly, the sample sizes of the completed clinical trials were relatively small, which may limit the generalizability of the findings. Secondly, the majority of the trials were conducted in high‐income countries, raising concerns about the applicability of the findings to patients in low‐ and middle‐income countries with different healthcare systems and resources. Thirdly, Warady and colleagues' trial it had a strong bias towards selection of the reported results because of the very high dropout rate in the bardoxolone‐arm as compared to the low drop‐out in the placebo arm. Lastly, the trials included in this study focused on a limited range of therapeutic interventions, leaving the potential benefits of other treatments unexplored.

### 4.5 Recommendations for Future Research

Based on the findings and limitations of this review, several recommendations are merited for future research:

1. Conduct larger, multi‐center clinical trials to improve the generalizability of findings and enable a more comprehensive assessment of the comparative effectiveness of different therapeutic interventions.
2. Include more diverse patient populations, with a focus on low‐ and middle‐income countries, to better understand the applicability of the findings across different healthcare settings and resource availability.
3. Investigate the potential benefits of other treatment options, such as anti‐fibrotic agents, protein translation modulators, and immunomodulatory agents, as well as combination therapies.
4. Assess the long‐term effects of these interventions on patient outcomes, including kidney function, quality of life, and survival.
5. Regularly update systematic reviews and meta‐analyses to incorporate recently published and ongoing studies, ensuring a comprehensive understanding of the current state of AS treatment research.

## 5 Conclusion

This systematic review provides preliminary evidence supporting the use of bardoxolone methyl, ramipril, and losartan in the treatment of AS. These findings align with existing literature and suggest that early initiation of these therapies could help preserve kidney function, potentially slow the progression of renal failure and thereby prevent multi‐system failure. However, the limited available evidence highlights the need for larger, more diverse clinical trials to confirm these findings and explore the potential benefits of other therapeutic interventions. Future research ought to focus on addressing these gaps and expanding our understanding of the most effective treatments for AS patients, ultimately improving patient outcomes and QoL.

## Author Contributions

**Zouina Sarfraz:** conceptualization, investigation, writing – original draft, methodology, writing – review and editing, formal analysis, data curation. **Ayesha Khan:** conceptualization, writing – original draft, writing – review and editing, visualization. **Maryyam Liaqat:** writing – original draft, writing – review and editing, methodology, data curation. **Aden Khan:** writing – original draft, writing – review and editing, methodology, data curation. **Faheem Javad:** conceptualization, writing – original draft, writing – review and editing, visualization. **Meher Saleem:** methodology, validation, writing – review and editing. **Azza Sarfraz:** conceptualization, writing – original draft, writing – review and editing, methodology. **Musfira Khalid:** conceptualization, writing – original draft, writing – review and editing, visualization. **Muzna Sarfraz:** conceptualization, writing – original draft, writing – review and editing; visualization. **Manish Kc:** conceptualization, writing – original draft, writing – review and editing, visualization. **Omar Irfan:** investigation, supervision, project administration, writing – review and editing.

## Ethics Statement

This study analyzes existing literature, does not involve primary data collection/interaction with human participants, and therefore, is exempt from obtaining ethical approval.

## Conflicts of Interest

The authors declare no conflicts of interest.

## Transparency Statement

The lead author Manish Kc affirms that this manuscript is an honest, accurate, and transparent account of the study being reported; that no important aspects of the study have been omitted; and that any discrepancies from the study as planned (and, if relevant, registered) have been explained.

## Supporting information

Supporting information.

## Data Availability

All data utilized for the purpose of this study is presented within the manuscript. All authors have read and approved the final version of the manuscript. Manish Kc had full access to all of the data in this study and takes complete responsibility for the integrity of the data and the accuracy of the data analysis.
